# Cardiovascular Benefits of Exercise Training in Postmenopausal Hypertension

**DOI:** 10.3390/ijms19092523

**Published:** 2018-08-25

**Authors:** Yi-Yuan Lin, Shin-Da Lee

**Affiliations:** 1 Graduate Institute of Clinical Medical Science, China Medical University, Taichung 40402, Taiwan; charlet8116@gmail.com.tw; 2 Department of Physical Therapy, Graduate Institute of Rehabilitation Science, China Medical University, Taichung 40402, Taiwan; 3 Department of Occupational Therapy, Asia University, Taichung 41354, Taiwan; 4 School of Rehabilitation Science, Shanghai University of Traditional Chinese Medicine, Shanghai 201203, China

**Keywords:** physical training, heart disease, cardiac remodeling, fibrosis, apoptosis

## Abstract

Exercise training is often considered the cornerstone of nonpharmacological therapy for postmenopausal hypertension while aerobic exercise is the mainstay of life style modification for antihypertension. Moderate-intensity aerobic exercise is well tolerated on most days of the week by most people with postmenopausal hypertension and is not suspected to detract from exercise adherence. That being said, moderate aerobic exercise may be superior for eliciting cardiovascular benefits in hypertensive postmenopausal women and resistance exercise may offer desirable benefits. The beneficial outcomes of exercise training for hypertensive postmenopausal women include improvements in blood pressure, autonomic tone, baroreflex sensitivity, oxidative stress, nitric oxide (NO), bioavailability, and lipid profiles, as well as cardiovascular function and cardiorespiratory fitness. This partly explains the fact that exercise training programs have a positive effect for cardiovascular disease in hypertensive postmenopausal women. This review is to collect and present the literature of exercise training in postmenopausal hypertension. Our review may provide the current understanding of beneficial effects and mechanisms of exercise intervention for prevention and treatment of stage 1 to 2 hypertensive postmenopausal women.

## 1. Introduction

In women after natural or surgical menopause, estrogen levels decrease gradually or drop suddenly. The menopause status in women can increase the risk of cardiovascular disease and metabolic disturbances. Especially, cardiovascular risks are more prevalent and highly influenced in those undergoing surgical menopause [1,2].

It is well known that hypertension is a potential risk factor for heart failure. Cross-sectional data show a fourfold increase in the incidence of hypertension occurring in postmenopausal women compared to in premenopausal women [3].This question is particularly important when hypertensive women reach the postmenopausal period, which may accelerate vascular aging and worsen cardiac dysfunction. The potential cardiovascular high risks after postmenopause are prone to the development of left ventricular hypertrophy, dilated cardiomyopathy, systolic dysfunction, cardiovascular remodeling, or higher cardiac output, which have been linked to the rapid increase in the risk of developing heart failure [4,5]. Hypertension in postmenopausal women is often associated with cardiovascular risk factors such as visceral obesity, dyslipidemias, chronic low-grade inflammation, oxidative stress, endothelial dysfunction, and cardiac remodeling. Therefore, there is a widespread need for treating and preventing cardiovascular risks in this high-risk population.

Many postmenopausal women took hormone replacement therapy (HRT) for many years to ease menopausal symptoms. However, long-term HRT is associated with an increased risk of breast cancer or severe side effects in some women, therefore, alternative therapeutic approaches need to be discovered for menopausal women [6,7]. Physical activity is an effective alternative to estrogen supplementation. Furthermore, aerobic exercise can significantly change estrogen metabolism, such as increase the ratio of the estradiol metabolites 2-hydroxyestrone and 16α-hydroxyestrone (2-OHE1/16α-OHE1) in premenopausal women, which may lower breast cancer risk [8]. Numerous studies have demonstrated the beneficial roles of exercise training in the cardiovascular system [9,10,11,12]. Regular physical activity is highly effective in improving aerobic fitness and physiological adaptations for cardiovascular health and reaches a 30–40% reduction in the risk of heart disease in all populations . It is well recognized that regular exercise training has been shown to be a therapeutic approach in managing cardiovascular diseases because it provides the most comprehensive benefits for reducing cardiovascular risk factors.

Since the underlying molecular mechanisms in hypertensive postmenopausal women is still unknown, the exercise-associated mechanisms for cardiac prevention will be interesting to follow. This review provides an update on the pathophysiological improvement of exercise training in stage 1–2 hypertensive postmenopausal women, suggests mechanisms by which exercise may improve cardiovascular function, and offers evidence-based recommendations regarding frequency, duration, and intensity of an exercise training program for stage 1 to 2 hypertensive postmenopausal women.

### 1.1. Relation between Body Mass Index, Exercise Training

Women’s weight gain after menopause is a well-recognized phenomenon which is accompanied by a multitude of changes in body composition [13]. Significant increase in the gain of body weight was observed in hypertensive rats after ovariectomy [14,15,16]. Epicardial fat is positively associated with obesity and body mass index (BMI). Myocardial steatosis and accumulation of fat in epicardial adipose tissue enhance the development of insulin resistance and type II diabetes mellitus, hypertension, and dyslipidemia, all of which also lead to more cardiovascular risk in women after menopause [13,17,18]. Exercise training showed a significant effect on BMI, fat mass, lean mass, visceral adipose tissue area, adiponectin level, serum lipids, and metabolic status [19,20]. One study showed that when obese (BMI range of 30 to 36 Kg/m^2^), hypertensive, postmenopausal women underwent eight weeks of walking training on an electric treadmill program (at least 20 min/session, 3 times/week, moderate intensity, with 60–70% of maximum heart rate), it led to a reduction of approximately −6% in BMI [21]. Another study showed that treadmill aerobic exercise training consisting of 24 sessions, 3 times/week and workload at maximal lactate steady state (MLSS) for eight weeks slightly reduced BMI approximately −1.5% in hypertensive postmenopausal women [22]. Nevertheless, 12 weeks of combined aerobic and resistance exercise training (60 min/session, 3 times/week, with 40–70% of heart rate reserve) did not change BMI, but body fat was decreased 2.12% and lean body mass was increased 0.81% in postmenopausal women with hypertension [23], indicating that increased muscle strength is inversely related with the risk of hypertension and frailty in older women [24,25]. However, it is still unknown whether exercise training following weight loss or change in body composition is associated with changes in the visceral adipose tissue and adiponectin levels under hypertensive postmenopausal status. Future studies that determine the effect of exercise training interventions on visceral adipose tissue and adiponectin levels are warranted.

### 1.2. Antihypertensive Effects of Exercise Training by Cardiovascular Autonomic Regulation

Physical activity is a common lifestyle modification and is recommended as first-line or adjunctive therapy. Several studies show that hypertensive postmenopausal women who engage in moderate-intensity physical activity, even through aquatic exercise training, experience a reduction in blood pressure (BP) [26,27,28].

Previous studies report that eight weeks of aerobic physical exercise reduce systolic BP, diastolic BP, and heart rate in hypertensive postmenopausal women approximately −3.8%, −5.9%, and −3.9%, respectively [22]. Aerobic or resistance exercise is known to effectively reduce blood pressure, which is associated with the improved cardiovascular autonomic mechanisms in hypertensive postmenopausal state [15,29,30]. A previous study reported that eight weeks of low-to-moderate- intensity exercise (50–60% maximal running speed) can enhance cardio-metabolic benefits, which are related to reduced blood pressure and autonomic dysfunction in hypertensive rats submitted to ovarian hormone deprivation [15]. Beneficial effects of aerobic exercise can be extended to ovariectomized hypertensive rats, as evidenced by arterial pressure reduction associated with enhanced cardiac vagal tonus and baroreflex sensitivity [15]. On the other hand, eight weeks of dynamic moderate-intensity resistance training diminished arterial pressure and heart rate in ovariectomized hypertensive rats due to a decrease in sympathetic tone [30]. Recently, evidence showed either resistance or aerobic exercise training is able to decrease arterial pressure and heart rate and to attenuate autonomic dysfunction, however, at different magnitudes and by different predominant mechanisms [31]. Moreover, the increase in baroreflex sensitivity after resistance or aerobic exercise training may be the mechanism involved in reduced arterial pressure. However, aerobic-trained groups had higher resting bradycardia than resistance-trained groups, associated with increase in parasympathetic modulation (RMSSD), which may account for a greater arterial pressure reduction. Additionally, the aerobic exercise training led to an increase in spontaneous baroreflex sensitivity (α index) rather than resistance exercise training [31]. Accordingly, these studies suggest the effectiveness of exercise training in lowering blood pressure in patients with uncontrolled hypertension. Moreover, higher levels of fitness could delay or prevent the development of arterial hypertension or its complications, indicating the beneficial effects of exercise training with regard to cardiovascular disease [29].

On the other hand, several studies suggest that exercise-associated improvement of cardiovascular function is associated with improvement of lipid profiles, antioxidative, nitric oxide bioavailability, cardiac fibrosis, and cardiac remodeling in hypertensive postmenopausal status.

### 1.3. Effect of Exercise Training on Lipid Profiles

A number of changes occurring in the lipid profiles during menopause are associated with increased metabolic and cardiovascular risks [32]. The lipid profiles under hypertensive postmenopausal status show intermediate levels: total cholesterol 214.15 mg/dL (194–261.87 mg/dL), high-density lipoprotein (HDL) 46.66 mg/dL (42.36–50 mg/dL), low-density lipoprotein (LDL) 132.49 mg/dL (124.7–152.36 mg/dL), and triglyceride (TG) 151.12 mg/dL (113.8–246.23 mg/dL) [22,28,29,33,34]. Endothelial dysfunction is also affected by a number of abnormal lipid parameters. Regular aerobic exercise has been shown to improve lipid abnormalities and endothelial function. A study of a group of fifteen women who were at least two years postmenopause and had a blood pressure range of 140/90–160/100 mmHg and BMI range of 25–31 Kg/m^2^, and who underwent three months of aerobic walking training on a treadmill program (30 min/session, 3 times/week, moderate intensity, with 60–75% of maximum heart rate) showed a significant increase in HDL of approximately 29.96%, and reduction in LDL of approximately –27.01%, and in TG approximately –17.70% [34]. Similarly, one study showed that 11 hypertensive postmenopausal women who underwent a six-month, moderate-intensity cycle ergometer training program (60 min/session, 3 times/week, moderate intensity, with 50% of heart rate reserve) reduced cholesterol levels by approximately –20% (initial: 220 ± 38, final: 178 ± 22) [33]. However, eight weeks of treadmill aerobic exercise training and twelve weeks of aquatic aerobic training resulted in no change in lipid profiles in postmenopausal hypertensive women [22,28]. The variation in the findings among these studies may be due to the differences in the type of the exercise regimens. In sum, training activities for at least 3 months of moderate-intensity exercise training can affect the lipid profiles. However, despite the same aerobic exercise frequency, the aquatic walk type of exercise did not impact the lipid profile.

We need to make a cautious note that in almost all studies, all the participants still received their needed drugs, but the studies compared those participating in the supervised combined aerobic exercise training program to a control group only receiving routine medical management [34]. A superior effect of combined aerobic exercise, with a greater increase in HDL and a greater decrease in LDL, triglycerides, and cholesterol in the combined exercise group when compared to the medicine-only group, indicated that medicine combined with exercise training may have a therapeutic advantage over monotherapy.

### 1.4. Antioxidative or Anti-Inflammatory Effects of Exercise Training

Increased oxidative stress is a common feature in the pathogenesis of arterial hypertension and postmenopause. Excessive oxidative stress has been observed in rat myocardium and coronary endothelial dysfunctions in hypertensive ovariectomy, suggesting that these impaired mechanisms may reflect a worsening heart failure in hypertensive postmenopausal women [14,31]. Reactive oxygen species (ROS) are implicated in the pathogenesis of cardiovascular disease such as hypertension, atherosclerosis, myocardial infarction, and cardiac failure. ROS can directly damage the lipids of cell membranes, proteins, and both nuclear and mitochondrial DNA, all of which result in serious or mortal cellular injury. However, several studies have demonstrated that vasodilation could be enhanced indirectly after exercise training by a distinct mechanism to increase antioxidant biomarkers such as superoxide dismutase (SOD) and catalase activities [14,35]. In human studies, eight weeks of treadmill aerobic exercise training promoted a profound increase in SOD activity in about 86.6% of hypertensive postmenopausal women [22]. The same intervention from Novais et al. reported that with eight weeks of exercise training in the trained hypertensive menopausal group, aerobic training was effective in promoting an increase in antioxidant biomarkers such as SOD (97%) and catalase (37%), which play a crucial role in oxidative stress modulation. In animal studies, Claudio et al. showed that eight weeks of interval training programs significantly enhanced mitochondrial SOD and catalase expression, and reduced cardiac superoxide production in rats with ovariectomized hypertension, which may prevent coronary heart disease in hypertensive postmenopausal women [14]. On the other hand, one study investigated how resistance training vs. aerobic training differentially impacts oxidative stress in ovariectomized hypertensive rats. The study reported that myocardial nicotinamide adenine dinucleotide phosphate (NAD(P)H) oxidase was 34.7% lower in the aerobic group compared with the ovariectomized hypertensive group and 31.1% lower in relation to the resistance training groups. Furthermore, concentration for the myocardial catalase was elevated in the aerobic training and resistance training groups when compared with the sedentary ovariectomized hypertensive group (117% and 72%, respectively) with an additional increment in the aerobic training group of approximately 20% when compared with the resistance training group, which indicated that aerobic-trained groups presented additional improvement when compared with resistance-trained groups, suggesting a better oxidative status after aerobic exercise over resistance exercise training [31]. These observations suggest that exercise training could contribute to improving excessive oxidative stress enhanced by postmenopausal hypertension. Moreover, the aerobic training group had a great and effective impact.

A growing body of evidence supports that elevated inflammation is involved in the initiation and progression of cardiovascular disorders. The effects of exercise training on inflammation under hypertensive postmenopausal status are still limited. Only one area of research determined the effects of exercise on inflammatory markers in hypertensive postmenopausal women. The study examined eight weeks of treadmill aerobic exercise training consisting of a maximal lactate steady state program on endocrine–inflammatory mediators in hypertensive postmenopausal women. Collectively, their findings show that endocrine–inflammatory mediators such as cortisol, leptin, and IL-1β did not contribute to the beneficial effects of the exercise training on blood pressure in hypertensive postmenopausal women [22]. Anti-inflammatory evidence of exercise training on hypertensive postmenopausal status is still too weak to draw any conclusions.

### 1.5. Antihypertensive Effects of Exercise Training by Improved NO Bioavailability and Vasorelaxation

Much evidence indicates that endothelial dysfunction plays a pivotal role in the initiation, development, and progression of several cardiovascular diseases. Impaired nitric oxide (NO) bioavailability is attributed to high oxidative stress in vascular endothelium, and it has been suggested to lead to the reduction in vasorelaxation and elevation in blood pressure by various causes under hypertensive condition. Vessels from ovariectomized hypertensive rats exhibit blunted arterial vasorelaxation in response to endothelium-dependent vasodilators such as acetylcholine (ACh) and bradykinin [14,35]. Furthermore, adverse aortic wall remodeling (wall thickness increase and smooth muscle cell hypertrophy) was enhanced in genetically hypertensive rats after ovariectomy [16]. However, this phenomenon can be effectively restored by increased physical activity and exercise training through the amelioration in mechanical and functional properties of the vasculature. Previous studies report that eight weeks of treadmill aerobic exercise training at maximal lactate steady state program significantly increased plasma nitrogen oxide (NOx) levels, which reflect nitric oxide (NO) production, as well as cyclic guanosine monophosphate (cGMP) concentration, approximately 37.7% and 30.8%, respectively, in hypertensive postmenopausal women [22]. A study consistently showed that a program of eight weeks of moderate-intensity aerobic exercise at 60–70% of maximum heart rate significantly increased plasma concentrations of nitrate/nitrite (NOx) by approximately 30.4% and improved systolic blood pressure (SBP) by approximately −16.2% [21]. Similarly, six months of bike aerobic training significantly increased plasma concentrations of nitrite/nitrate levels by approximately 60% and improved blood pressure SBP by approximately −12.7% in postmenopausal women with hypertension [33]. Furthermore, twelve weeks of combined aerobic and resistance exercise training can increase blood nitrate/nitrite (NOx) approximately Δ28%, and improved blood pressure SBP by approximately −8% in postmenopausal women with hypertension [23]. Aerobic exercise training seems to have greater improved BP and enhancement of nitrate/nitrite (NOx) than combined aerobic and resistance exercise training, which reflects NO production is positively correlated with duration of training, and negatively correlated with BP variability in postmenopausal hypertensive women.

These increased levels of NO after exercise training may enhance endothelial-dependent dilation, improve arterial stiffness, reduce vascular resistance, and decrease vascular tone in peripheral arteries and consequently may contribute to reductions in BP [23]. Indeed, the beneficial effects of exercise training on blood pressure were related to an improvement of the NO/cGMP-mediated vasorelaxation [22]. Previous studies have shown that exercise training corrects endothelial function by enhancing ACh and bradykinin-induced, endothelium-dependent vasorelaxation through the endothelial NOS (eNOS)/NO signaling pathway in ovariectomized hypertensive animals [14,35].

**Figure 1:**
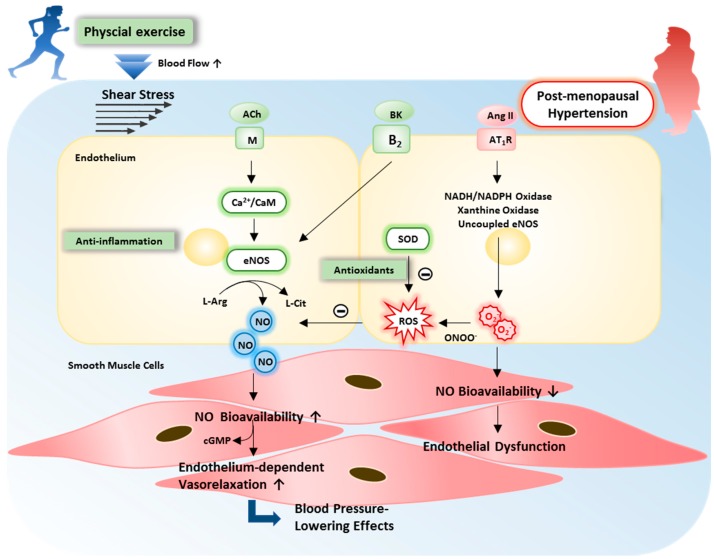
The potential mechanisms of exercise training on vascular endothelial dysfunction in postmenopausal hypertension. The mechanisms initiating endothelial dysfunction involve the oxidative stress, inflammation, and impaired reflect nitric oxide (NO) bioavailability and vasorelaxation in postmenopausal hypertension. Among many ROS, increases in nicotinamide adenine dinucleotide phosphate (NAD(P)H) oxidase, xanthine oxidase, and uncoupled endothelial NOS (eNOS) are associated with hypertension. Exercise training evokes the improvements in anti-inflammation, antioxidant production, NO bioavailability, and endothelium-dependent vasorelaxation. This partly explains the blood-pressure-lowering effects of exercise in postmenopausal hypertension. Specifically, the exercise-induced shear stress and activation of various receptors (e.g., M, B_2_) lead to the increases in NO production and bioavailability by inducing eNOS expression in the endothelium. ↑: up-regulation; ↓: down-regulation.

Physiologically, exercise-induced cardiac output is increased and likely augments shear stress in vessels where blood flow is increased. The exercise-induced increase in shear stress has been regarded as a significant mechanism for NO production in vivo, which is a highly effective and sensitive system to counteract myogenic and neurogenic-induced vascular contraction during exercise [36,37,38]. Exercise preserves NO bioavailability in vascular endothelium, which has been proven with evidence via increasing NO production and upregulated eNOS phosphorylation and its enzyme activity [36]. Until now, exactly how exercise training improves NO bioavailability and vascular function is still unclear in hypertensive postmenopausal women. However, the exercise-induced shear stress provides one of the major mechanisms for improvements of NO bioavailability and vascular endothelial function in the exercise-induced cardiovascular protection. As seen in Figure 1, we summarized and proposed the underlying mechanisms of exercise-induced shear stress for improving endothelial dysfunction.

### 1.6. Cardiac Remodeling, Antifibrosis, or Antiapoptotic Effect of Exercise Training

Myocardial adverse remodeling through the oxidative stress and fibrous tissue accumulation contribute significantly to the development of heart failure [39]. Because collagen deposition contributes to the development of cardiac adverse remodeling and fibrosis, reduction of collagen plays a key role in reducing adverse remodeling [40,41]. Pathologic growth in heart wall thickness and volume is associated with cell death (apoptosis or necrosis) and often progresses to severe cardiac remodeling and heart failure leading to functional decompensation, whereas physiologic growth is associated with a normal or increased cardiac function [39]. Recent studies have reported that cardiac abnormalities in ovariectomized hypertensive rats have demonstrated increased cardiomyocyte hypertrophy, myocardial interstitial space, and reparative fibrosis, and more cardiomyocyte apoptosis [16,42].

**Figure 2:**
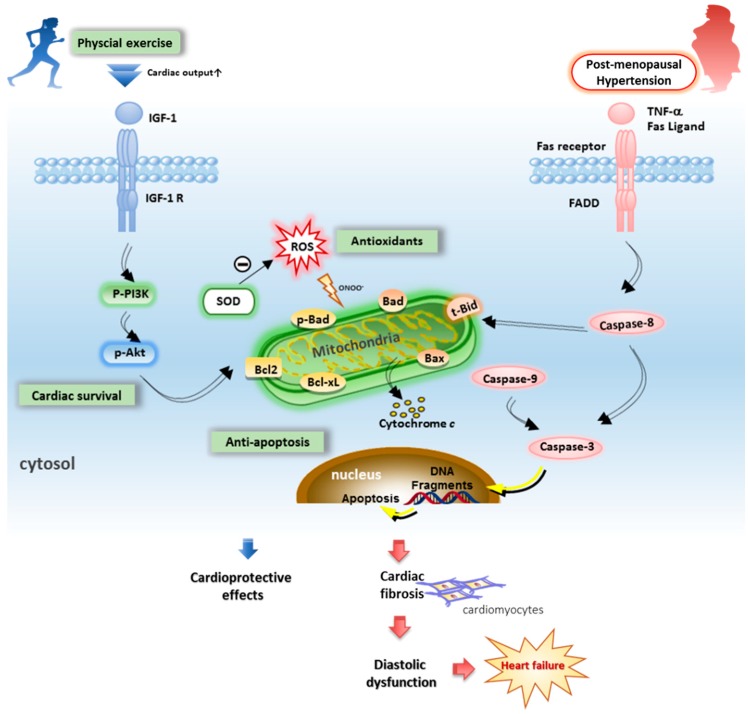
Hypothesized exercise training may improve pathophysiology of heart failure in postmenopausal hypertension. The mechanisms initiating diastolic dysfunction involve the oxidative stress and Fas- and mitochondria-dependent apoptotic pathways in postmenopausal hypertension. Among many of reactive oxygen species (ROS), increases in NAD(P)H oxidase are associated with postmenopausal hypertension. Exercise training evokes the improvements in antioxidant production and cardiac survival, and suppresses cardiac widely dispersed apoptosis. This partly explains the cardioprotective effects of exercise in postmenopausal hypertension. blue arrows: induced cardioprotective effects; red arrows: induced myocardial dysfunction effects.

Lifestyle changes and regularly performed physical activity can counteract defects in associated heart failure processes. Aerobic exercise training is a well-known benefit adaptive to the cardiovascular system. A study conducted by Almeida et al. shows that exercise training attenuated interstitial collagen and myocyte hypertrophy, which improved cardiac functioning and attenuation of cardiac remodeling in ovariectomized rats after myocardial infarction [43]. Our previous studies also reported that exercise training can significantly improve cardiac apoptosis in hypertension [44] and ovariectomies [45]. Exercise training prevented hypertension-induced cardiac Fas-dependent and mitochondria-dependent apoptotic pathways via enhanced cardiac insulin-like growth factor 1 receptor (IGFI-R)/phosphoinositide 3-kinase (PI3K)/protein kinase B (Akt) and Bcl-2-family-associated prosurvival pathways in hypertension [44]. Moreover, estrogen deprivation by ovariectomy displays cardiac Fas-dependent and mitochondria-dependent apoptotic pathways which appear to be improved after exercise training [45]. One report suggests that exercise training exerts beneficial effects which in turn diminish adverse cardiac wall remodeling, mainly by reducing interstitial myocardial fibrosis, improving myocardial vascularization, and sustaining the number of cardiomyocytes [16]. These findings revealed that either hypertension or ovariectomy after exercise training provides beneficial effects on cardiac function and cardiac remodeling by the altered regulation of the specific gene and protein expression that regulate myocardial apoptosis and fibrosis. Until now, the influences and molecular mechanisms of resistance training or combined resistance and endurance training in the hypertensive, postmenopausal population remain unclear and need further investigation. After integrating previous findings into hypothesized pathophysiology, we hypothesize that exercise training may suppress cardiac Fas- and mitochondrial-dependent apoptotic pathways in the coexistence of hypertension and ovariectomy. Apoptotic cadiomyocyte and accumulated collagens can also contribute to the development of cardiac fibrosis and heart failure (Figure 2).

### 1.7. Aerobic Training vs. Resistance Training for Postmenopausal Hypertension

In sum, moderate aerobic exercise may be superior for eliciting cardiovascular benefits in hypertensive postmenopausal women including improvements in blood pressure, autonomic tone, baroreflex sensitivity, oxidative stress, NO bioavailability, lipid abnormality, cardiovascular function, and cardiorespiratory fitness. Furthermore, moderate aerobic exercise has a greater effect on increasing parasympathetic modulation, baroreflex sensitivity, NO bioavailability, antioxidative ability, and dyslipidemia improvement than resistance exercise. Nevertheless, resistance exercise may offer desirable benefits such as increased lean body mass and increased muscle strength.

It is well known that regular exercise can have a substantial positive effect on various cardiovascular conditions. Pretty much any type of “aerobic” exercise will be beneficial. Aerobic exercise should be emphasized, with some resistance exercise included, although we observed aerobic exercise training seemed to have greater improvement on blood pressure and enhancement of NOx than combined aerobic and resistance exercise training in postmenopausal hypertensive women. However, increased muscle strength is inversely related with the risk of hypertension and frailty in older women [24,25]. A beneficial aspect of combined exercise may be reduced arterial stiffness and blood pressure in postmenopausal women with hypertension, indicating that combined exercise modality may be clinically beneficial for reducing the risk of frailty and mortality in hypertensive postmenopausal women [23].

The new information from the 2017 American College of Cardiology/American Heart Association update of high blood pressure was reported [46]. Normal BP remains the same as in JNC 7 (average SBP < 120 mm Hg and average diastolic blood pressure (DBP) < 80 mm Hg); “prehypertension” with “elevated BP” is defined as an average SBP: 120–129 mm Hg and average DBP < 80 mm Hg; “stage 1 hypertension” is defined as an average SBP: 130–139 mm Hg or average DBP: 80–89 mm Hg; and “Stage 2 hypertension“ is defined as an average SBP ≥ 140 mm Hg or an average DBP ≥ 90 mm Hg. We need to make a cautious note that all participants were hypertensive postmenopausal women whose blood pressure average range was SBP: 117–152 mm Hg and DBP 73–95 mm Hg. Moreover, nearly all studies didn’t exclude drug therapy, indicating that all participants still needed drug therapy. Nevertheless, it would also imply that combined exercise training may have a therapeutic advantage over monotherapy [34].

Table 1 and Table 2 summarized the effects of exercise training on human and animal hypertensive postmenopausal studies.

## 2. Conclusions

This review is to briefly examine and summarize the recent evidence regarding the clinical and experimental effects of aerobic or resistance exercise training on cardiovascular risk factors in hypertensive postmenopausal women.

Among individuals who are hypertensive postmenopausal, all types of “aerobic” exercise training programs have a positive effect for cardiovascular disease. The beneficial outcomes of exercise training programs include improvements in blood pressure, autonomic tone, baroreflex sensitivity, oxidative stress, NO bioavailability, lipid profiles, cardiovascular function, and cardiorespiratory fitness. Further research could be focused on the integration of aerobic and resistance exercise for the improvement of cardiovascular function and molecular mechanisms in hypertensive postmenopausal women, such as anti-inflammation, visceral adipose tissue and adiponectin levels, and antifibrotic or antiapoptosis signaling pathways. Additionally, clinical and experimental studies are required to clarify the possible therapeutic applications through different exercise interventions in hypertensive postmenopausal women.

This review suggest that various forms of endurance and resistance exercise may have a beneficial effect on hypertensive postmenopausal women. Overall, the studies reviewed herein support the therapeutic concept to promote physical activity and to achieve physical fitness, and the essential conclusion is that moderate aerobic exercise may be superior for eliciting cardiovascular benefits in hypertensive postmenopausal women and resistance exercise may offer desirable benefits.

## Figures and Tables

**Table 1:** Summary of exercise training protocols and outcomes from postmenopausal hypertensive women.

Study	Age	Initial SBP/DBP	Number	Training Type	Training Intensity	Training Frequency	Outcomes
Staffileno et al., 2001 [27]	57 ± 9 years	150/94 mm Hg	pre: 9 post: 9	Walk	Moderate (50–60% HRR)	10min/time, 3 times/day, 5 days/week, 8 weeks	SBP ↓
Latosik et al., 2014 [26]	N/A	146/85 mm Hg	pre: 15 post: 15	Walk	N/A	8 weeks	SBP ↓ Maximal aerobic capacity ↑
Ammar 2015 [34]	52–53 years	152/94 mm Hg	pre: 15 post: 15	Walktreadmill	Moderate (60–75% MHR)	3 months	SBP, DBP ↓ LDL, TG, cholesterol ↓ HDL ↑
Zaros et al., 2009 [33]	50 ± 4 years	141/90 mm Hg	pre: 11 post: 11	Cycle ergometer	Moderate (50% HRR)	60 min/session, 3 times/week, for 6 months	SBP, DBP ↓ Heart rate ↓ Cholesterol ↓ Nitrite/Nitrate (NOx-) ↑
Khalid et al., 2013 [21]	53 ± 3 years	148/94 mm Hg	pre: 18 post: 18	Walktreadmill	Moderate (60–70% MHR)	Least 20 min/session, 3 times/week, for 8 weeks	SBP, DBP ↓ BMI ↓ Nitrite/Nitrate (NOx-) ↑
Jarrete et al., 2014 [22]	58 ± 1 years	117/73 mm Hg	pre: 28 post: 28	Walk	MLSS	30–40 min/session, 3 times/week, for 8 weeks	SBP, DBP ↓ Heart rate ↓ Nitrite/Nitrate (NOx-) ↑ cGMP ↑ SOD ↑
Novais et al., 2017 [29]	57 ± 1 years	117/73 mm Hg	pre: 28 post: 28	Walk	MLSS	30–40 min/session, 3 times/week, for 8 weeks	cGMP ↑ SOD, Catalase ↑
Arca et al., 2014 [28]	64 ± 7 years	136/85 mm Hg	pre: 19 post: 19	Aquaticwalk	Moderate (50–60% HRR)	50 min/session, 3 times/week, for 12 weeks	SBP ↓
Son et al., 2017 [23]	75 ± 2 years	145/95 mm Hg	pre: 10 post: 10	Combined resistance and aerobic training	Moderate (40–70% HRR)	70min/day, 3 times/week, for 12 weeks	SBP, DBP and MAP ↓ ET-1 ↓ Functional capacity ↑ V̇O_2_max ↑ Nitrite/Nitrate (NOx-) ↑ baPWV ↑

MHR: Maximum heart rate; HRR: Heart rate reserve; MLSS: maximal lactate steady state; SBP: Systolic blood pressure; DBP: Diastolic blood pressure; MAP: Mean arterial pressure; LDL: Low-density lipoprotein, HDL: High-density lipoprotein, TG: Triglyceride; cGMP: cyclic guanosine monophosphate; SOD: Superoxide dismutase; V̇O_2_max: maximal oxygen consumption; baPWV: brachial-ankle pulse wave velocity; N/A: not describe; ↑: up-regulation; ↓: down-regulation.

**Table 2:** Summary of exercise training protocols and outcomes from ovariectomized hypertensive animals model.

Study	Age	Number	Training Type	Training Intensity	Training Frequency	Results
Marques et al., 2006 [16]	14 weeks	Sed-int: 7 Sed-ovx:7 Ex-int: 7 Ex-ovx: 7	Treadmill	Low	60 min/day, 5 days/week, for 13 weeks	SBP ↓ Aortic wall thickness ↓ Cardiac fibrosis ↓ Vascularization ↑ Cardiomyocyte number ↑
Sanches et al., 2012 [15]	14 weeks	SHO:7 THO: 7	Treadmill	Low- moderate(~50–60% maximal running speed)	60 min/day, 5 days/week, for 8 weeks	SBP, DBP and MAP ↓ Body weight ↓ Vagal tone ↑ Baroreflex sensitivity ↑
Da Palma et al., 2016 [31]	13 weeks	HS: 8 HSO: 8 HATO: 8 HRTO: 8	Treadmill	Low-moderate(~50–60% maximal running speed)	60 min /day, 5 days/week, for 8 weeks	SBP, DBP and MAP ↓ Heart rate ↓ VAR-PI, RMSSD ↑ Baroreflex sensitivity ↑ NADPH oxidase, Lipid peroxidation, protein oxidation ↓ Catalase, SOD, GSH ↑
Da Palma et al., 2016 [31]	13 weeks	HS: 8 HSO: 8 HATO: 8 HRTO: 8	Resistance	Moderate	5 days/week, 8 weeks	SBP, DBP and MAP ↓ Heart rate ↓ RMSSD ↑ NADPH oxidase, Lipid peroxidation, protein oxidation ↓ SOD, GPx ↑
Shimojo et al., 2015 [30]	13 weeks	SC: 8 SH: 8 SHO: 8 RTHO: 8	Resistance	Moderate	5 days/week, 8 weeks	MAP ↓ Heart rate ↓ Sympathetic tone ↓
Claudio et al., 2017 [14]	13 weeks	SH: 12 SSW:13 OVX: 13 OSW: 13	Swimming	N/A	60 min/day, 5 days/week, for 8 weeks	Heart rate ↓ Cardiac superoxide produce ↑ Vasodilation ↑ Arteries SOD, catalase ↑

MHR: Maximum heart rate; HRR: Heart rate reserve; SBP: Systolic blood pressure; DBP: Diastolic blood pressure; MAP: Mean arterial pressure; VAR-PI: Pulse interval variability, RMSSD: root-mean-square of successive differences, SOD: Superoxide dismutase; GPx: guaiacol peroxidase. SC: sedentary normotensive group. Sed-int, HS, SH: sedentary hypertensive rats group. Sed-ovx, SHO, HSO, OVX: ovariectomized hypertensive rats group. Ex-int, SSW: hypertensive rats plus aerobatic exercise training. Ex-ovx, THO, HATO, OSW: ovariectomized hypertensive rats plus aerobatic training. HRTO, RTHO: ovariectomized hypertensive rats plus resistance training; N/A: not describe; ↑: up-regulation; ↓: down-regulation.
